# Phycova — a tool for exploring covariates of pathogen spread

**DOI:** 10.1093/ve/veac015

**Published:** 2022-02-18

**Authors:** Tim Blokker, Guy Baele, Philippe Lemey, Simon Dellicour

**Affiliations:** Department of Microbiology, Immunology and Transplantation, Rega Institute, KU Leuven, Herestraat 49, Leuven 3000, Belgium; Department of Microbiology, Immunology and Transplantation, Rega Institute, KU Leuven, Herestraat 49, Leuven 3000, Belgium; Department of Microbiology, Immunology and Transplantation, Rega Institute, KU Leuven, Herestraat 49, Leuven 3000, Belgium; Spatial Epidemiology Lab. (SpELL), Université Libre de Bruxelles, CP160/12, 50, av. FD Roosevelt, Bruxelles 1050, Belgium

**Keywords:** discrete phylogeography, covariates, generalized linear model, linear regression, BEAST, PhyCovA, visualization, pathogen spread

## Abstract

Genetic analyses of fast-evolving pathogens are frequently undertaken to test the impact of covariates on their dispersal. In particular, a popular approach consists of parameterizing a discrete phylogeographic model as a generalized linear model to identify and analyse the predictors of the dispersal rates of viral lineages among discrete locations. However, such a full probabilistic inference is often computationally demanding and time-consuming. In the face of the increasing amount of viral genomes sequenced in epidemic outbreaks, there is a need for a fast exploration of covariates that might be relevant to consider in formal analyses. We here present PhyCovA (short for ‘Phylogeographic Covariate Analysis’), a web-based application allowing users to rapidly explore the association between candidate covariates and the number of phylogenetically informed transition events among locations. Specifically, PhyCovA takes as input a phylogenetic tree with discrete state annotations at the internal nodes, or reconstructs those states if not available, to subsequently conduct univariate and multivariate linear regression analyses, as well as an exploratory variable selection analysis. In addition, the application can also be used to generate and explore various visualizations related to the regression analyses or to the phylogenetic tree annotated by the ancestral state reconstruction. PhyCovA is freely accessible at https://evolcompvir-kuleuven.shinyapps.io/PhyCovA/ and also distributed in a dockerized form obtainable from https://hub.docker.com/repository/docker/timblokker/phycova. The source code and tutorial are available from the GitHub repository https://github.com/TimBlokker/PhyCovA.

## 1. Introduction

Phylogeographic analyses (Lemey et al. 2009, 2010; Müller et al. 2018) are frequently used in molecular epidemiology to investigate the drivers of spatial spread of fast-evolving pathogens, such as RNA viruses (Lemey et al. 2014; Müller et al. 2019; Dellicour et al. 2020). In particular, adopting a generalized linear model (GLM) in phylogeographic reconstruction has become a popular approach to test the association of the transition rates between discrete locations (e.g. countries) and a series of potential predictors or covariates (Lemey et al. 2014). This approach has, for instance, been used to study the predictors of Ebola virus (EBOV) spread during the 2014–2016 epidemic in West Africa (Dudas et al. 2017). Based on the analysis of >1,600 EBOV genomes, this study highlighted that viral lineages tend to preferentially disperse between geographically closer and highly populated locations. Implemented in the software package BEAST 1.10 (Suchard et al. 2018), this GLM approach is, however, associated with a relatively high computational burden related (1) to the need to perform the GLM analysis while averaging over many plausible evolutionary histories and (2) to the number of distinct parameters to be estimated in such joint Bayesian phylogeographic inference (involving estimation of parameters for the coalescent, substitution, and molecular clock models, as well as for the discrete diffusion model used for the phylogeographic reconstruction and associated GLM).

During the COVID-19 pandemic, an unprecedented amount of viral genomes have been sequenced and made publicly available (∼8 × 10^6^ SARS-CoV-2 genomes deposited on GISAID in February 2022; https://www.gisaid.org/). To support large-scale phylogeographic reconstructions, it would be useful to rapidly explore dispersal rate predictors that would be relevant to include in such analyses. To fill this gap, we here present PhyCovA, a novel user-friendly application that can be used to quickly investigate a (potentially large) series of predictors of spatial spread. The development of PhyCovA has been inspired by applications such as TempEst (Rambaut et al. 2016), which was developed to perform an exploratory investigation of the temporal signal in a data set of time-stamped sequences, a prerequisite for the subsequent calibration of a molecular clock model to infer time-calibrated phylogenetic trees. Similar to TempEst, PhyCovA is a software tool that can be used to perform exploratory analysis prior to more formal and more time-consuming probabilistic inferences to identify the drivers of viral spread.

## 2. Design and implementation

**Figure 1.**
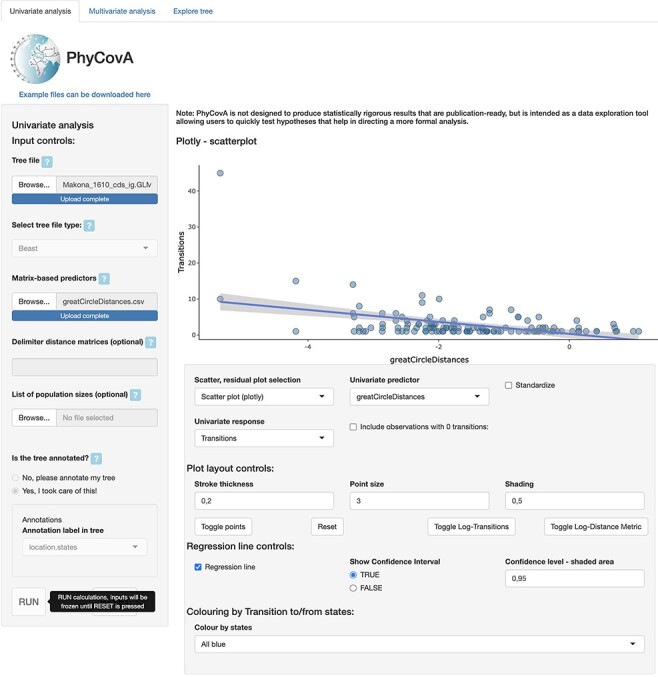
User interface of the PhyCovA online application. On the left-hand side, the annotated phylogeny along with the potential predictors of pathogen spread can be uploaded to the application. On the right-hand side, a scatter plot explores the association (or lack thereof) between the transition rates and a selected predictor.

PhyCovA, short for ‘Phylogeographic Covariate Analysis’, has been developed as a browser-based application. The baseline code has been written in R using the package ‘shiny’ (https://shiny.rstudio.com/) to make PhyCovA accessible as a web application with a graphical user interface (Fig. 1). The application allows the user to explore which predictors (e.g. geographic distance, air traffic, and population size at the location of origin/destination) tend to correlate with the number of viral lineage transitions among locations. Those transition counts are either extracted from an annotated phylogenetic tree, i.e. a tree for which the location of ancestral nodes has already been estimated or from a tree for which ancestral reconstruction of internal nodes still has to be performed. In the latter case, the ancestral reconstruction can be performed as a first analytical step in PhyCovA by using either a maximum-likelihood method implemented in the R package ‘ape’ (Paradis and Schliep 2019) or a maximum parsimony method implemented in the R package ‘castor’ (Louca and Doebeli 2018). In both cases, i.e. if the tree was previously annotated or if it needs to be annotated in PhyCovA, a tree traversal is performed to count the number of lineage transitions between locations, leading to an asymmetric matrix of these pairwise transition counts. Specifically, transition events are identified by comparing the locations assigned to the parent and child nodes connected by phylogenetic branches: a transition event is inferred when the location assigned to a child node is different from the location assigned to its parent node in the tree.

In addition to a rooted phylogenetic tree with or without annotations, PhyCovA requires the user to load the following input files: a matrix or several matrices to be tested as potential predictor(s) (e.g. geographic distances among locations, a binary metric specifying if each pair of locations share a specific administrative border), a list of location-specific values of interest to be tested as potential predictors (e.g. population density, economic metrics, or measures of averaged climatic variables at each location), and an ordered list of tip locations (only when loading a tree without state annotations).

PhyCovA has three tabs that support different tasks (Fig. 1). First, the ‘Univariate analysis’ tab allows for univariate regression analysis and serves, at the same time, as the user interface to upload the different input files and specify the ancestral reconstruction method (if the provided phylogenetic tree is not already annotated; see above). The choices made in the univariate tab before clicking ‘RUN’ are the only inputs that are not reactive in PhyCovA. All other inputs are reversible and can be changed or tuned. Upon clicking ‘RUN’, the ‘Univariate analysis’ tab provides the user with a scatter plot and associated linear regression, as well as other optional graphs (a scatter plot of the linear regression residuals, a barplot reporting the total number of transition events to/from each location). Below the graphical elements, the ‘Univariate analysis’ tab can also detail the results of the univariate linear regression analysis. The second tab, ‘Multivariate analysis’, allows selecting predictors for analysis in a multivariate linear regression model. The different predictors can be selected using interactive tick-boxes in the first panel, and data transformation (log-transformation and standardization) can be carried out by the user. The results of the multivariate linear regression analysis and associated graphs (e.g. scatter plot, correlation matrix) are reported in the two subsequent panels. Multivariate analyses may include results from a variable selection analysis, e.g. based on the Bayesian information criterion and performed with the ‘regsubsets’ function of the R package ‘leaps’ (https://cran.r-project.org/web/packages/leaps/index.html). The third and last tab in PhyCovA (‘Explore tree’) uses functions from the R package ‘ggtree’ (Yu et al. 2017) to offer the user the possibility to visualize and explore the annotated tree. Further, users of PhyCovA may be interested in the visualization of transition events between countries, which can be readily performed by the SPREAD (Bielejec et al. 2011) and spreaD3 (Bielejec et al. 2016) software packages, which were specifically developed for mapping the dispersal history of viral lineages on geographic maps.

We note that there are important differences between the linear regression approach used in PhyCovA and Bayesian phylogeographic inference that makes use of a GLM. Linear regression enables estimating a linear relationship between a dependent variable and one or more explanatory (or independent) variables. In classical statistics, observed data are typically used as the variables of interest. In phylogenetics, however, the dependent variable is typically an estimable parameter. The linear regression approach we use here first estimates the dependent variable — the number of lineage transitions along an annotated phylogeny, in the case of PhyCovA — in a phylogenetic framework and subsequently uses those estimates in a standard linear regression approach (Rambaut et al. 2016). As a result, uncertainty related to the phylogeny or the lineage transition events is not taken into account. The GLM generalizes linear regression by allowing to model the response variable through a link function (e.g. log, identify, and inverse) and allowing the magnitude of the variance of each measurement to be a function of its predicted value. It unifies various other statistical models, including linear regression, logistic regression, and Poisson regression (Zhao 2012). The GLM approach (Lemey et al. 2014), as implemented in BEAST 1.10 (Suchard et al. 2018), parameterizes the transition rates between locations as a log-linear function of a set of explanatory (or independent) variables, typically called predictors or covariates. The coefficient for each predictor is estimated throughout the BEAST analysis, as well as its inclusion probability, allowing to estimate the contribution and support for each predictor while accommodating phylogenetic (and parameter) uncertainty. This procedure also enables estimating transition events between locations by means of Markov jumps (Minin and Suchard 2008) but albeit using a time-consuming approach, especially when analysing high-dimensional data sets.

Similar to the regression of sampling time against root-to-tip genetic distance used to investigate temporal signal in the program TempEst (Rambaut et al. 2016), the linear regressions implemented in PhyCovA are not suitable for hypothesis testing because we do not assess if regression residuals are normally distributed and associated with a constant variance (homoscedasticity). Consequently, the coefficient of determination (R^2^) and associated p-value are not valid statistical estimates (Drummond et al. 2003; Rambaut et al. 2016). In addition, several sources of estimation uncertainty are ignored. For these reasons, PhyCovA is not designed to produce statistically rigorous results that are publication-ready, but it is intended as a data exploration tool allowing users to quickly investigate hypotheses that may inform a more formal analysis.

## Availability

PhyCovA is freely accessible online at https://evolcompvir-kuleuven.shinyapps.io/PhyCovA/ and also distributed in a dockerized form obtainable from https://hub.docker.com/repository/docker/timblokker/phycova. The source code is publicly available at https://github.com/TimBlokker/PhyCovA/, and the application tutorial can be found at https://github.com/TimBlokker/PhyCovA/blob/master/tutorial/PhyCovA_Tutorial.pdf.
